# Thoracic spinal epidural hematoma misdiagnosed as conversion paralysis: A case report

**DOI:** 10.1002/ccr3.8434

**Published:** 2024-01-29

**Authors:** Tadatsugu Morimoto, Hirohito Hirata, Takuya Nikaido, Kenichiro Taniguchi, Tomohito Yoshihara, Takaomi Kobayashi, Masatsugu Tsukamoto, Masaaki Mawatari

**Affiliations:** ^1^ Department of Orthopedic Surgery, Faculty of Medicine Saga University Saga Japan; ^2^ Department of Orthopaedic Surgery Fukushima Medical University Fukushima Japan; ^3^ Saga Yebisu Mental Clicic Saga Japan

**Keywords:** conversion paralysis, misdiagnosis, SIC test, spinal epidural hematoma

## Abstract

A woman with a history of psychiatric hospitalization was misdiagnosed with conversion paralysis despite lower extremity paralysis due to a thoracic epidural hematoma, leaving her with severe neurological deficits. Conversion paralysis is a diagnosis of exclusion and should never be made unless all possible organic causes have been ruled out.

## Graphical abstract

Sagitta T2‐weighted Magnetic resonance imaging (MRI) (Left) MRI before hematoma removal showed spinal cord epidural hematoma at the T8‐L1 level. (Right) MRI at 3 weeks after hematoma removal: hematoma was removed, but spinal cord degeneration was evident.

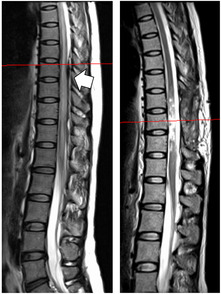

## 1 INTRODUCTION

The diagnosis of conversion paralysis (CP) or hysterical paralysis (HP) is sometimes challenging as a diagnosis of exclusion for the clinician.^1^, ^2^, ^3^, ^4^ The prevalence of patients with conversion palsy admitted to spinal cord units has been noted to be 0.3%–3.8%.^2^, ^5^, ^6^, ^7^, ^8^

Although women are often misdiagnosed with CP, they may have genuine spinal cord lesions.^1^ In such cases, failure to “diagnose and treat,” especially in a timely manner, can result in an irreversible neurological damage that could have been avoided.^1^ Therefore, it is very important to keep in mind that a diagnosis of CP should never be made unless all possible organic causes have been ruled out.^8^

Here we report a case of a woman who developed paralysis of both lower limbs and was misdiagnosed as CP. A few days later, she was diagnosed with thoracic spinal cord epidural hematoma (SCEH) and underwent emergency hematoma removal surgery but remained severely paralyzed.

## 2 CASE REPORT

### 2.1 Case history and examination

A 48‐year‐old woman visited the emergency room in the early morning because of sudden‐onset muscle weakness in both lower limbs after defecating. She had a history of psychiatric illness (alcohol use disorder) and hospitalizations for said concern but had no history of CP, hypertension, or stroke. The patient had a history of alcoholism and alcohol liver disorder. She was taking anti‐alcoholic medication and had not consumed alcohol for the past year. The patient was not on any anticoagulants or antiplatelet medications.

Physical examination revealed bilateral lower extremity muscle weakness (manual muscle test 0–1), hypesthesia, loss of patellar tendon and Achilles tendon reflexes, and negative Babinski reflexes. Blood tests showed no abnormalities in coagulation capacity, platelet count, electrolytes, or liver and kidney function. No imaging studies were performed. The patient was able to maintain a seated position in the clinic. Due to the patient's history of psychiatric visits, this led to a diagnosis of CP. The patient was admitted to the psychiatric hospital where she had been previously admitted.

**FIGURE 1:**
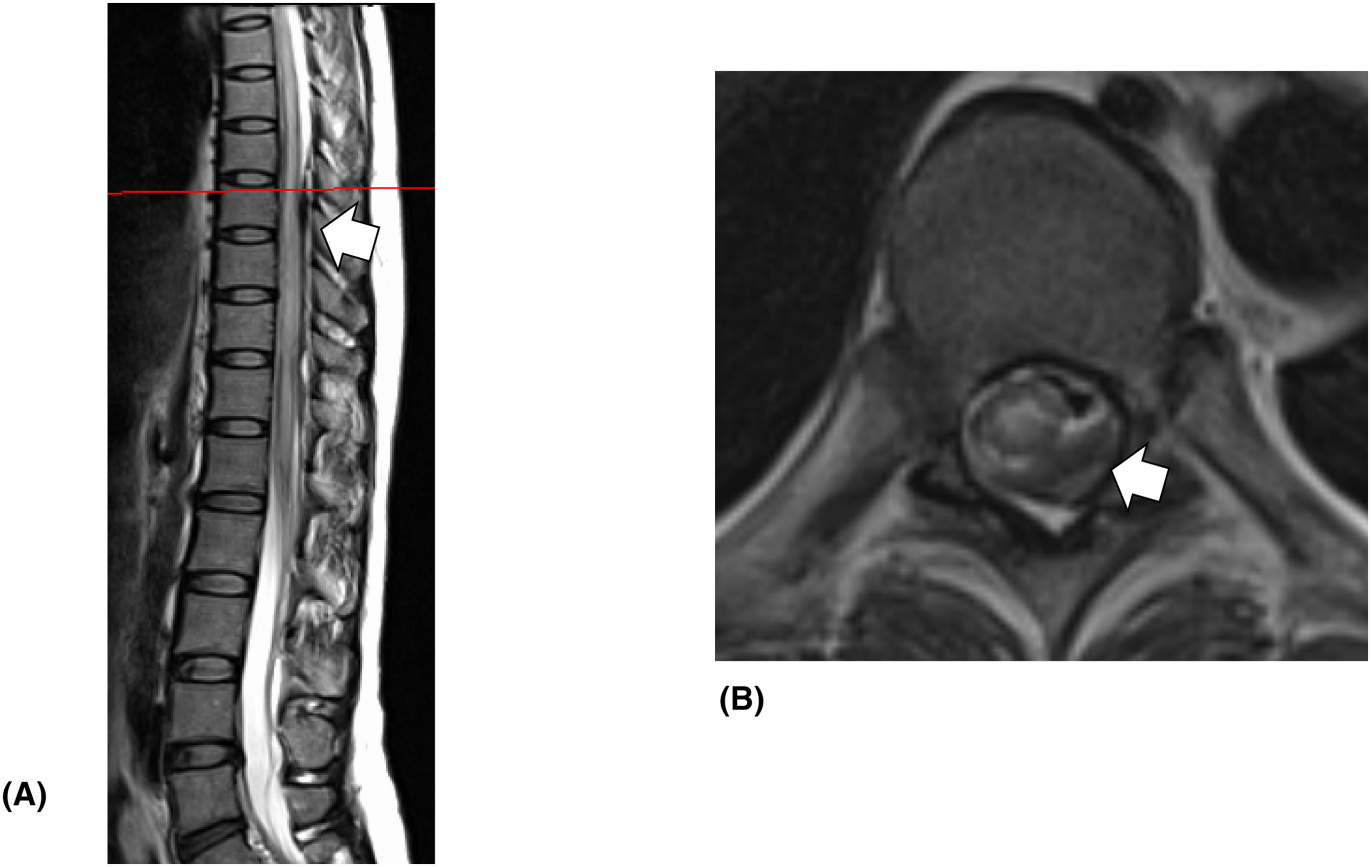
Magnetic resonance imaging (MRI) before surgery. (A) Sagittal T2‐weighted MRI showing spinal cord epidural hematoma at the T8–L1 level, with T9 displaying the area with the most severe spinal cord compression (white arrow) and extensive spinal cord edema. (B) Axial T2‐weighted MRI showing significant left‐sided spinal cord epidural hematoma at T9 level.

After 4 days, her paralysis still was not resolved, and she underwent thoracolumbar magnetic resonance imaging (MRI) (Figure 1) at the psychiatric hospital where she was transferred. A diagnosis of spinal cord epidural hematoma (SCEH) and spinal cord degeneration at the thoracic level was made, and the patient was transferred to our hospital.

### 2.2 Treatment, outcome, and follow‐up

Due to the persistent complete motor paralysis and hypesthesia (Frankel B) of the patient below the level examined, she underwent emergent hematoma removal. Unfortunately, the patient's severe paralysis persisted.

**FIGURE 2:**
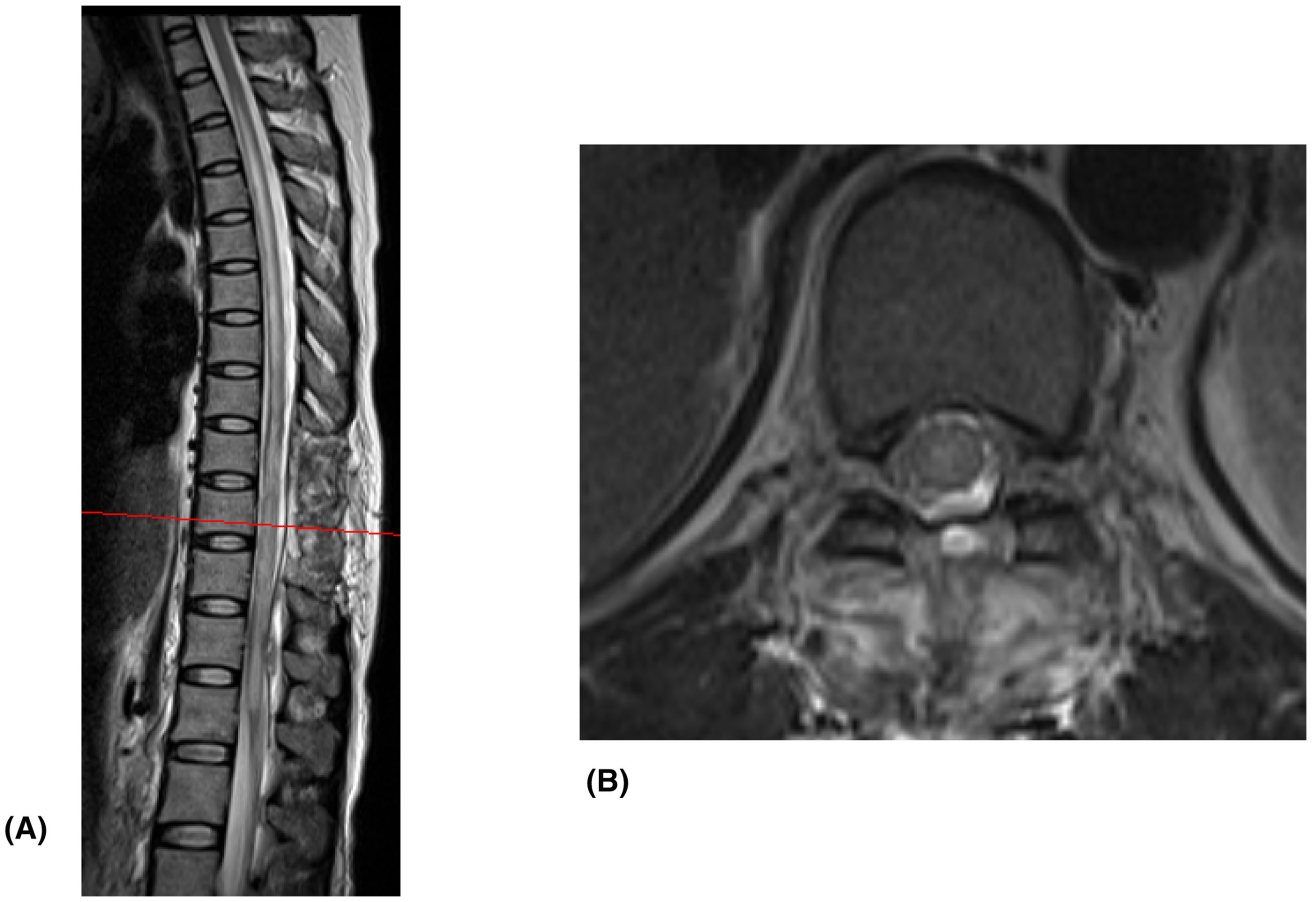
Magnetic resonance imaging (MRI) 3 days after surgery. (A) Sagittal and axial. (B) T2‐weighted MRI showing postoperative findings after laminectomy and hematoma removal, and decompression of the thoracic spinal cord. Extensive spinal cord edema (T2 to the conus medullaris) remains.

MRI on postoperative day 3 confirmed removal of the lesion around T9, which had the most compression on the spinal cord due to the hematoma (Figure 2).

**FIGURE 3:**
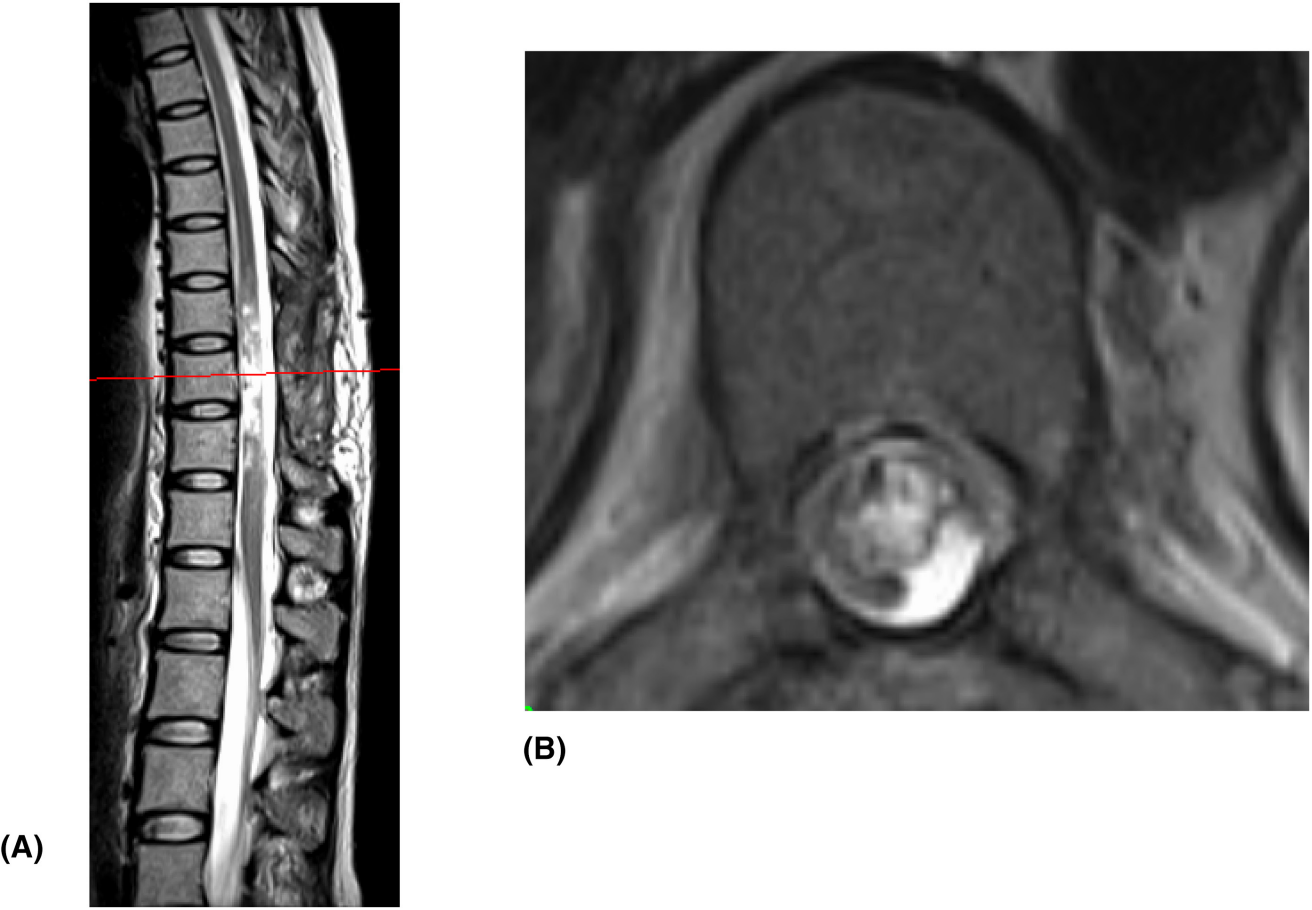
Magnetic resonance imaging (MRI) at 3 weeks after surgery. (A) Sagittal and axial. (B) T2‐weighted MRI showing resolution of the hematoma, but there remains an irregular, well‐defined hyperintense signal in the T9‐11 levels.

However, the patient's paraplegia did not resolve after the surgery. MRI done at 3 weeks postoperatively showed complete resolution of the hematoma, but there was evident spinal cord degeneration (Figure 3).

One year after surgery, the patient remains paralyzed (Frankel B).

## 3 DISCUSSION

### 3.1 Clinical feature of spontaneous SCEH

The clinical course of spontaneous SCEH often begins with neck or back pain in the case of cervical or thoracic epidural hematoma, respectively, followed by a transition to spinal symptoms.^1^, ^9^, ^10^, ^11^ Symptoms of SCEH are more likely to appear with activities and states that increase intravascular pressure can cause bleeding, such as pregnancy, exertion, or straining (i.e., Valsalva maneuver).^10^, ^12^, ^13^ Predisposing factors for SCEH include a history of coagulopathy, use of anticoagulant or antiplatelet medications, hematologic disorders, tumors of the spinal cord, hypertension, and alcoholism.^14^, ^15^ Interestingly, hemorrhagic spinal complications, including SCEH, have been one of the most common spinal cord lesions caused by SARS‐CoV‐2 invasion.^16^, ^17^ Alcoholism and alcoholic liver disorders have been noted to cause hemostatic disturbances, resulting in bleeding from even minor events.^18^, ^19^ This patient was taking anti‐alcoholic medication and did not consume any alcohol for the past year, and blood tests showed no abnormalities in coagulation capacity or liver function; therefore, the effects of alcohol were unknown, although they may have been latent.

Poor prognostic factors for SCEH include the onset of pain at the mid and lower thoracic back, use of anticoagulants, loss of sphincter tone, severe neurologic impairment on admission, short progression interval, and spinal cord edema on MRI.^20^, ^21^ SCEH has a time‐sensitive prognosis, with delayed diagnosis potentially leading to permanent neurological sequelae and death. As a rule, emergency hematoma removal is recommended for patients with severe neurologic dysfunction or clinical deterioration.^20^, ^21^ Early hematoma removal within 12–24 h is the key to improving patient outcomes.^12^, ^13^, ^22^, ^23^

This patient had a thoracic epidural hematoma that caused sudden‐onset motor paralysis of both lower limbs after defecation, followed thereafter by a severe spinal cord injury. Although emergency hematoma removal was warranted, the delay in diagnosis resulted in severe neurological damage.

### 3.2 Differentiation of CP

This case is a typical example of the pitfalls of mental health bias or mental illness‐related stigma,.^24^ in which physical symptoms are mistakenly attributed to psychiatric causes without conducting a thorough physical examination because the patient had a history of psychiatric illness (alcohol use disorder).

CP remains a diagnosis of exclusion, requiring a systematic and thorough approach. Although electrophysiological diagnostics (motor and somatosensory evoked potentials) and fMRI help clinch the diagnosis, these diagnostics may not be widely available, and cost may be an issue.^8^

The neurological examination is important in clinical practice. Barre's sign, Hoover's test, and the Spinal Injuries Center (SIC) test are some unique clinical tests that can be done to detect CP.^8^, ^25^

Barre's sign places the patient in a prone position, with their knees flexed at right angles. The examiner then releases the support of the knees and instructs the patient to maintain that position; in true paralysis, the affected limb will extend.^8^, ^25^ In Hoover's test, the patient is in supine position, while the examiner lifts both of the patient's legs slightly off of the exam table.^8^, ^25^ The patient is asked to lift the affected limb, while the examiner supports both limbs from the heel. If there is no heel downward pressure on the contralateral limb (non‐affected limb), the patient indicates apathy of intent.^8^, ^25^ However, Barre's sign and Hoover's test—first reported in 1919 and 1908, respectively—the classic famous clinical tests for the evaluation of hemiplegia are useless for evaluating paraplegia.^8^, ^25^

The SIC test was designed to evaluate patients with CP and lower extremity motor deficits. The patient lies in a supine position, while the physician passively lifts the patient's knee to the flexed position and places the foot flat on the bed. The physician holds the patient's knees apart. The test is considered positive if the patient can maintain the flexed position.^8^ By contrast, the patient cannot maintain the knee flexed in severe paralysis, and the paralyzed leg spontaneously falls into flaccid extension. In this case, the SIC test can be considered negative. The SIC test is very simple to conduct and is noninvasive, yet it can make the correct diagnosis of CP (100% sensitivity and 97.9% specificity).^8^ The SIC test is an examination technique all physicians should be familiar with. It can be useful in making an accurate bedside diagnosis of hysterical paralysis versus a more severe etiology of paralysis.

Despite their profession, medical providers may still hold a “mental health‐related stigma” and believe that patients' symptoms are attributable to their psychiatric illness.^24^ Being aware of these cognitive biases, a diagnosis of CP should never be made unless a systematic and thorough evaluation has ruled out all possible organic causes.

## 4 CONCLUSION

Spontaneous SCEH is a disease for which prompt and accurate diagnosis is crucial; a delay in diagnosis can lead to permanent neurological sequelae. Patients should not be labeled with CP until adequate physical findings and ancillary studies, such as imaging and electrophysiological studies, have ruled out an organic cause. The SIC test is a simple, noninvasive test that can differentiate CP from true/severe paralysis and should be known not only by spine surgeons and neurologists but also by primary care and family physicians.

## AUTHOR CONTRIBUTIONS

**Tadatsugu Morimoto:** Conceptualization; data curation; writing – original draft; writing – review and editing. **Hirohito Hirata:** Conceptualization; formal analysis; writing – original draft. **Takuya Nikaido:** Formal analysis; methodology; validation; writing – review and editing. **Kenichiro Taniguchi:** Conceptualization; supervision; validation; writing – review and editing. **Tomohito Yoshihara:** Data curation; validation; visualization; writing – original draft. **Takaomi Kobayashi:** Data curation; validation; writing – original draft. **Masatsugu Tsukamoto:** Formal analysis; supervision; writing – review and editing. **Masaaki Mawatari:** Investigation; methodology; supervision; writing – review and editing.

## FUNDING INFORMATION

None.

## CONFLICT OF INTEREST STATEMENT

The authors declare that they have no competing interests.

## ETHICS STATEMENT

Not mandated for case reports.

## CONSENT

Written informed consent was obtained from the patient and the patient's family to publish this report in accordance with the journal's patient consent policy.

## Data Availability

Data pertaining to this case can be obtained by contacting the corresponding author

## References

[1] Epstein NE . Review/perspective on hysterical paralysis: a diagnosis of exclusion for spinal surgeons. Surg Neurol Int. 2022;13:172. doi:10.25259/SNI_278_2022 35509596 PMC9062961

[2] Letonoff EJ , Williams TR , Sidhu KS . Hysterical paralysis: a report of three cases and a review of the literature. Spine (Phila Pa 1976). 2002;27(20):E441‐E445. doi:10.1097/01.BRS.0000029268.16070.D8 12394915

[3] Watanabe TK , O'Dell MW , Togliatti TJ . Diagnosis and rehabilitation strategies for patients with hysterical hemiparesis: a report of four cases. Arch Phys Med Rehabil. 1998;79(6):709‐714. doi:10.1016/s0003-9993(98)90049-1 9630154

[4] Weingarden SI , Lynch CG . Functional paralysis mimicking spinal cord injury resulting in admission to a spinal cord injury center. Arch Phys Med Rehabil. 1984;65(3):145‐147. 6703891

[5] Heruti RJ , Levy A , Adunski A , Ohry A . Conversion motor paralysis disorder: overview and rehabilitation model. Spinal Cord. 2002;40(7):327‐334. doi:10.1038/sj.sc.3101308 12080460

[6] Heruti RJ , Reznik J , Adunski A , Levy A , Weingarden H , Ohry A . Conversion motor paralysis disorder: analysis of 34 consecutive referrals. Spinal Cord. 2002;40(7):335‐340. doi:10.1038/sj.sc.3101307 12080461

[7] Maurice‐Williams RS , Marsh H . Simulated paraplegia: an occasional problem for the neurosurgeon. J Neurol Neurosurg Psychiatry. 1985;48(8):826‐831. doi:10.1136/jnnp.48.8.826 4031935 PMC1028456

[8] Yugué I , Shiba K , Ueta T , Iwamoto Y . A new clinical evaluation for hysterical paralysis. Spine (Phila Pa 1976). 2004;29(17):1910‐1913. doi:10.1097/01.brs.0000137055.55350.37 15534415

[9] Mohamed EH , Dsouza LB , Elnabawy WA , Bashir K , Elmoheen A . Acute spinal extradural hematoma and cord compression: case report and a literature review. Cureus. 2020;12(11):e11603. doi:10.7759/cureus.11603 33240731 PMC7681751

[10] Shima H , Yasuda M , Nomura M , et al. A spinal epidural hematoma with symptoms mimicking cerebral stroke. Nagoya J Med Sci. 2012;74(1–2):207‐210. 22515129 PMC4831268

[11] Morimoto T , Yoshihara T , Yakushiji Y , et al. Worsening cervical epidural hematoma after tissue plasminogen activator administration for stroke like symptoms. Spine (Phila Pa 1976). 2016;41(7):E437‐E440. doi:10.1097/BRS.0000000000001243 26693669

[12] Nakanishi K , Nakano N , Uchiyama T , Kato A . Hemiparesis caused by cervical spontaneous spinal epidural hematoma: a report of 3 cases. Adv Orthop. 2011;2011:516382. doi:10.4061/2011/516382 21991415 PMC3170783

[13] Hu Y , Su J , Cui X , Pan L , Jin L , Teng F . How to avoid misdiagnosing spontaneous cervical spinal epidural hematoma as ischemic stroke: 3 case reports and literature review. Cerebrovasc Dis. 2023;52(5):597‐606. doi:10.1159/000527705 36516738

[14] Peng D , Yan M , Liu T , et al. Prognostic factors and treatments efficacy in spontaneous spinal epidural hematoma: a multicenter retrospective study. Neurology. 2022;99(8):e843‐e850. doi:10.1212/WNL.0000000000200844 35715197 PMC9484729

[15] Sheng OC , Wu RC , Chang IH . Spontaneous spinal epidural hematoma: a case report. Int J Emerg Med. 2021;14(1):60. doi:10.1186/s12245-021-00379-0 34563108 PMC8466974

[16] Sourani A , Vahdat N , Son C , et al. SARS‐CoV‐2 infection and spontaneous spinal hemorrhage: a systematic review. Neurosurg Rev. 2023;46(1):300. 37966587 10.1007/s10143-023-02211-0

[17] Sourani A , Rezvani M , Foroughi M , Baradaran MS . Spontaneous intramedullary hematoma following COVID‐19 vaccination: A case report. Clin Case Rep. 2022;10(12):e6743. 36545562 10.1002/ccr3.6743 PMC9761661

[18] Shoji K , Miyagawa N , Tanikawa A , Kobayashi M . Retropharyngeal hematoma in a patient with chronic alcoholism. Am J Emerg Med. 2021;46:799.e793‐799.e794. 10.1016/j.ajem.2021.01.087 33558096

[19] Mangla A , Hamad H , Yadav U , Telfer M . Alcohol abuse and alcoholic liver cirrhosis leading to spontaneous muscle hematoma: an event fraught with danger. Case Rep Gastroenterol. 2015;9(1):93‐100. 25969676 10.1159/000381305 PMC4427135

[20] Huang D , Iken S , Elbadri S , Falgiani M , Ganti L . Spontaneous Spinal Epidural Hematoma: A Case of a Benign Presentation and Emergency Department Management. Cureus. 2022;14(3):e23532. 35494915 10.7759/cureus.23532 PMC9040688

[21] Bakker NA , Veeger NJ , Vergeer RA , Groen RJ . Prognosis after spinal cord and cauda compression in spontaneous spinal epidural hematomas. Neurology. 2015;84(18):1894‐1903. 25862799 10.1212/WNL.0000000000001545

[22] Rajz G , Cohen JE , Harnof S , et al. Spontaneous spinal epidural hematoma: the importance of preoperative neurological status and rapid intervention. J Clin Neurosci. 2015;22(1):123‐128. 25156033 10.1016/j.jocn.2014.07.003

[23] Shin JJ , Kuh SU , Cho YE . Surgical management of spontaneous spinal epidural hematoma. Eur Spine J. 2006;15(6):998‐1004. 16758110 10.1007/s00586-005-0965-8 PMC3489451

[24] Knaak S , Mantler E , Szeto A . Mental illness‐related stigma in healthcare: Barriers to access and care and evidence‐based solutions. Healthc Manage Forum. 2017;30(2):111‐116. 28929889 10.1177/0840470416679413 PMC5347358

[25] Smith HE , Rynning RE , Okafor C , et al. Evaluation of neurologic deficit without apparent cause: the importance of a multidisciplinary approach. J Spinal Cord Med. 2007;30(5):509‐517. 18092568 10.1080/10790268.2007.11754585 PMC2141729

